# Systematic Review and Meta-Analysis of Liraglutide Treatment in Children Who Are Overweight or Obese: A Therapeutic Paradigm Shift?

**DOI:** 10.7759/cureus.83738

**Published:** 2025-05-08

**Authors:** Felipe A. Muñoz Rossi, Edison A Aristizábal, Kassen Saleh, Donovan A Sánchez, Néstor Israel Quinapanta Castro, Jonathan Coronel, Lina A Villota, Juan D. Gonzalez, David A Ibarra, Gina Paola Ricardo Ossio

**Affiliations:** 1 Methodology of Health Sciences Research, International University of La Rioja, Bogotá, COL; 2 Internal Medicine, National University of Colombia, Bogotá, COL; 3 Pediatrics, Universidad de Antioquia, Medellín, COL; 4 Pediatric Medicine, Universidad Simón Bolívar, Barranquilla, COL; 5 General Medicine, Universidad Catolica Santiago De Guayaquil, Guayaquil, ECU; 6 Research and Biostatistics, Universidad Regional Autónoma de los Andes (UNIANDES), Ambato, ECU; 7 Obstetrics and Gynecology, Hospital Materno Infantil José Domingo De Obaldía, David, PAN; 8 General Medicine, Universidad de Guayaquil, Guayaquil, ECU; 9 General Medicine, Universidad de Cartagena, Cartagena, COL; 10 General Medicine, Centro Especializado David, David, PAN; 11 Medical Affairs, Universidad del Rosario, Bogota, COL; 12 Clinical Research, Universidad Metropolitana de Barranquilla, Barranquilla, COL

**Keywords:** glp-1 receptor agonist, liraglutide, meta-analysis, overweight, pediatric obesity, systematic review

## Abstract

Pediatric obesity is a public health problem with long-term repercussions, in which there is limited effectiveness of interventions such as lifestyle changes. This study evaluated the efficacy and safety of liraglutide in overweight/obese children and adolescents (six to 18 years) using a systematic review and meta-analysis of randomized clinical trials (RCTs). Databases such as PubMed, Web of Science, and ClinicalTrials.gov were searched up to February 2025, and the analysis included four RCTs (n=378). Liraglutide significantly reduced BMI Z-score (standardized mean difference or SMD: -1.03; 95% CI: -1.24 to -0.81; I²=0%) and showed modest improvements in HbA1c (SMD: -1.14; 95% CI: -2.10 to -0.17; I²=92%), though with high metabolic heterogeneity. There was a tendency toward increased hypoglycemia (relative risk or RR: 1.55; 95% CI: 1.00-2.40), but there was no significant difference in the overall adverse effects (RR: 1.06; 95% CI: 0.97-1.15). The results support the use of liraglutide to reduce BMI in this population, but the current evidence is limited by the small number of studies, methodological biases, and variability in metabolic outcomes. More robust RCTs and studies with prolonged follow-up are needed to consolidate liraglutide's role in the management of pediatric obesity.

## Introduction and background

Pediatric obesity constitutes one of the most prevalent chronic conditions globally, with an estimated 107 million people suffering from obesity in 2020 [1,2], and affecting approximately 14.4 million children and adolescents [3]. In the United States, nearly 20% of children under 19 years of age suffer from obesity [4], while in Colombia, the prevalence among those under 18 years old is 17.53%, which translates to around 2.7 million cases [5]. Additionally, it is estimated that nearly 70% of the studied population will remain obese into adulthood, which generates a burden of chronic disease associated with metabolic syndrome and high cardiovascular risk, along with all the health consequences that are already widely known [1].

Obesity is often underestimated in its etiological complexity. In adults, it is usually reduced to a personal choice, but in the pediatric population, multiple causes such as genetic, metabolic, phenotypic, socioeconomic, and environmental have been identified. However, they do not fully explain the phenomenon or its pandemic spread, and additional factors such as urban environments and changes in physical activity patterns merit further investigation [6-8].

According to the WHO, one of the main causes of obesity is the low intake of nutrients, which is considered one of the greatest threats to public health [2]. In obese patients, an increase in inflammation and oxidative stress is observed at the level of adipose tissue and hypothalamus (with an elevation of tumor necrosis factor (TNF)-alpha, IL-1β, IL-6, and other cytokines). These effects are associated with hypercaloric diets that are poor in micronutrients and fat-soluble vitamins, which enhance oxidative damage and reduce the intestinal absorption of these nutrients [1,2].

This is compounded by factors such as the absence of breastfeeding, genetic syndromes (Prader-Willi, Turner, monogenic obesity, among others), and contemporary issues that affect metabolic health, such as sedentary lifestyle, eating disorders, poverty, limited access to healthy foods, bullying, racism, etc. [2]. In this multifactorial context, it is essential to adopt comprehensive treatments based on scientific evidence and tailored to the physiological needs of this population [9,10]. The etiology of childhood obesity is multifactorial; many of these individuals may have alterations in metabolic and epigenetic immune processes that increase the risk of obesity by disrupting energy regulation [11]. This influence is greater in children who are exposed to negative environmental and social determinants, such as racism [12], low socioeconomic status, and even immigration, as well as limited access to healthy food options, highlighting the importance of these negative social determinants in the etiology of those who suffer from overweight and obesity [7].

Childhood obesity is defined as a body mass index (BMI) greater than or equal to the 95th percentile for the age and sex, and severe obesity as a BMI greater than or equal to 120% of the 95th percentile for the age and sex [13]. Another measure used is the conversion of BMI to z-scores. This classification is based on the standard deviations (SD) relative to the mean of the reference population, generally defined by the World Health Organization (WHO), with a normal weight defined as a z-score between -2 and +1 SD, overweight ≥+2 SD and <3 SD, and obesity with a z-score ≥+3 SD [14]. This classification allows for the identification of cases and facilitates the implementation of early and effective interventions.

Liraglutide is an acylated human glucagon-like peptide-1 (GLP-1) receptor agonist that acts to increase insulin release in the presence of elevated glucose concentrations, thereby reducing the rate at which postprandial glucose appears in the circulation [15]. GLP-1 regulates appetite and caloric intake, even though its receptors are present in the brain. The weight reduction effect of liraglutide is due to the decrease in caloric intake and it has widely demonstrated its efficacy in the management of obesity in adults [16].

Childhood obesity represents a public health problem of epidemic proportions, with long-term implications for the physical and mental health of the pediatric population. The pharmacological management of childhood obesity was limited to orlistat and phentermine, but as adjuncts to lifestyle changes, and only limited to adolescents. In 2020, the FDA approved the use of liraglutide as a part of the pharmacological management of obesity but excluded the age group between six and 12 years, given the limited evidence at that time [17].

Although liraglutide shows significant metabolic benefits, its effectiveness in children and adolescents could be enhanced through a comprehensive approach that includes lifestyle modifications, such as promoting healthy eating and regular physical activity. In this way, the intervention would not only act on the physiological mechanisms involved in obesity and diabetes but also promote sustainable behavioral changes that maximize its effects and improve the quality of life of the patients. This systematic review and meta-analysis aims to synthesize the available evidence on liraglutide's efficacy and safety in pediatric patients, providing a solid foundation for clinical decisions and future research on childhood obesity.

## Review

Methods

This study aims to determine the efficacy of liraglutide, a GLP-1 receptor agonist, in reducing weight in overweight and obese children and adolescents. To this end, its efficacy and safety will be compared with other standard interventions, such as lifestyle modifications, behavioral therapy, and other drugs approved for the treatment of childhood obesity.

We conducted a systematic review with a meta-analysis of randomized controlled clinical trials (RCTs) evaluating the effect of liraglutide on weight reduction in children and adolescents. We included studies with participants aged six to 18 years diagnosed as overweight or obese, defined as a BMI greater than or equal to the 85th percentile for age and sex for overweight and greater than or equal to the 95th percentile for obesity, according to WHO or Centers for Disease Control and Prevention (CDC) growth charts. Participants had to have shown an inadequate response to previous interventions based on lifestyle modifications for at least six months.

Those with medical conditions that could affect weight, such as genetic syndromes associated with obesity, uncontrolled hypothyroidism, chronic corticosteroid use, diagnosis of type 1 or 2 diabetes mellitus on insulin therapy, history of acute or chronic pancreatitis, and known allergy or hypersensitivity to liraglutide or any of its components, were excluded.

Comparators included standard interventions based on lifestyle modifications, which consisted of structured programs combining hypocaloric diet, increased physical activity, and behavioral therapy. Drugs approved for the treatment of pediatric obesity according to current clinical guidelines, such as orlistat or metformin, in addition to a placebo control group, were also included. These comparators enabled the assessment of liraglutide’s relative efficacy and safety against current standard interventions in the pediatric population.

The primary outcomes analyzed were the change in BMI expressed as a z-score for age and sex, assessed up to 56 weeks of follow-up, and the percentage reduction in body weight from baseline to the end of the intervention period. Secondary outcomes included an improvement in metabolic parameters such as fasting glucose levels and HbA1c. The incidence of treatment-associated adverse effects, such as nausea, vomiting, pancreatitis, or hypoglycemia, as well as adherence to treatment and dropout rates, was also evaluated.

To identify studies, a systematic search strategy was performed across several electronic databases, including PubMed, Medline, and Web of Science, from their inception until February 2025. A controlled vocabulary based on Medical Subject Headings (MeSH) and free-text terms was used, considering spelling variants, synonyms, acronyms, and truncators. The search strategy used Boolean operators and restricted the language to Spanish and English. In addition, a search was conducted using other sources such as ClinicalTrials.gov and a manual review of the bibliographic references of the selected studies.

The identified studies were independently reviewed by the authors using the Rayyan platform. A selection process was carried out based on reading titles, abstracts, and then the full text, and disagreements were resolved by consensus. Data extraction was performed using a form specifically developed by our review team following guidance from the Cochrane Handbook [17], in which information was collected on the author, type of GLP-1 agonist, study duration, number of participants, year, country, study design, route of administration, study date, diagnostic criteria, inclusion and exclusion criteria, comparator characteristics, and study registry number.

The risk of bias in the included studies was independently assessed using the Cochrane tool for systematic reviews with intervention (RoB 2.0) [18]. The following domains were analyzed: random sequence generation, allocation concealment, blinding of participants and personnel, incomplete outcome data, and selective reporting. For the categorization of overall bias risk, the Cochrane criteria were employed [18], classifying the studies into low risk of bias, high risk of bias, uncertain risk, or intermediate risk. Disagreements were settled by consensus or by the ultimate opinion of the lead author.

The treatment effect measures included the calculation of the relative risk (RR) with 95% confidence intervals (CI) for dichotomous data. For continuous data, the mean difference (MD) was used when outcomes were measured uniformly across studies, and the standardized mean difference (SMD) was used in case of variations in the measures. For heterogeneity, the I² statistic was used, considering high heterogeneity values above 50%. For these cases, a random effects model is used as a strategy to minimize the variability between studies.

Publication bias was evaluated through funnel plots and the Egger test, although this analysis was only conducted if the number of included studies exceeded ten clinical trials. A description of the findings for each outcome was made, evaluating the quality of the evidence using the GRADE approach, considering factors such as risk of bias, inconsistency of results, indirect evidence, imprecision of results, and publication bias.

This study is registered in the International Prospective Register of Systematic Reviews (PROSPERO; CRD420251015493). The complete protocol, publicly available on that platform, details the objectives, selection criteria (inclusion/exclusion), systematic search strategies, and analytical methodology, thus ensuring methodological transparency and the reproducibility of the research.

Results

Search Results

**Figure 1:**
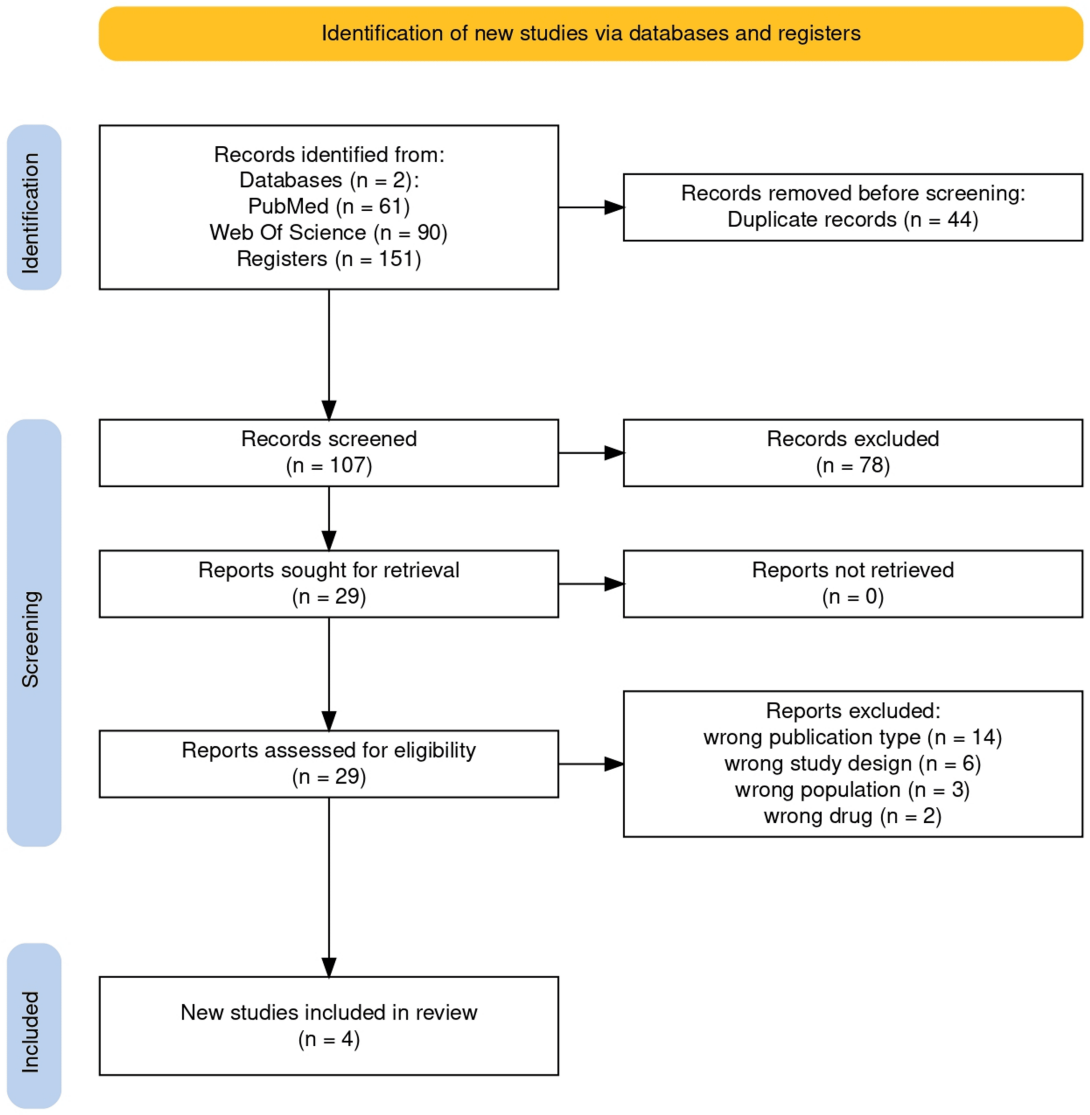
PRISMA flow diagram for systematic review

Our initial search identified 151 references across the selected databases. After removing duplicates (n=44) and screening titles/abstracts, we excluded 78 records. Of the 29 remaining articles assessed in full text, 14 were excluded due to publication type, six for study design, three for population mismatch, and two for being outside the review scope. Ultimately, four studies met all eligibility criteria and were included in the systematic review (see PRISMA flow diagram, Figure 1) [19].

Characteristics of the Included Studies

The studies included in this systematic review correspond to randomized, double-blind, placebo-controlled clinical trials conducted in different countries and in pediatric populations with obesity.

Danne et al. [20] conducted a study in Germany on adolescents aged 12 to 17 with a BMI equal to or above the 95th percentile, without a history of diabetes, to evaluate the safety and pharmacokinetics of liraglutide. Participants with a diagnosis of type 1 or type 2 diabetes or previous use of GLP-1 agonists were excluded.

Mastrandrea et al. [21] conducted a study in the U.S. on children aged seven to 11 years with obesity without a history of diabetes. Those with a diagnosis of type 1 or type 2 diabetes, Tanner stage greater than or equal to 2, or a history of pancreatitis were excluded.

Kelly et al. [22] conducted a phase 3 clinical trial in multiple countries (Belgium, Mexico, Russia, Sweden, and the USA) involving adolescents aged 12 to 18 with obesity who had not responded to lifestyle interventions. Participants with type 1 diabetes or those undergoing treatment with other obesity drugs were excluded.

Fox et al. [23] conducted a multicenter phase 3a study at 23 sites in nine countries in children aged six to less than 12 years with obesity and no history of diabetes. Those with prior use of obesity medications or pre-existing metabolic medical conditions were excluded.

In all the studies, the intervention consisted of subcutaneous administration of liraglutide in variable doses (0.3-3.0 mg/day or 3.0 mg/day), while the comparator groups received a placebo. The outcomes evaluated included changes in BMI, body weight, and the incidence of adverse events (Table 1).

**Table 1:** Characteristics of the included studies SC, subcutaneous; SD, standard deviation

Author	Study design	Country	Year	Objectives	Inclusion criteria	Exclusion criteria	Intervention	Comparator	Outcomes assessed	Main results	Conclusion
Danne et al., 2016 [20]	Randomized, double-blind, placebo-controlled clinical trial	Germany	2016	Assess the safety and pharmacokinetics of liraglutide in adolescents with obesity	12-17 years old, BMI ≥ p95, no diabetes	Type 1 or 2 diabetes, prior use of GLP-1 agonists	Liraglutide 0.6-3.0 mg/day SC	Placebo	BMI, adverse events	No significant changes in BMI, good tolerability	Liraglutide is safe in adolescents with obesity; further studies are needed
Mastrandrea et al., 2019 [21]	Randomized, double-blind, placebo-controlled clinical trial	USA	2019	Assess the safety and pharmacokinetics of liraglutide in children with obesity	7-11 years old, BMI ≥95th percentile, no diabetes	Type 1 or 2 diabetes, Tanner stage ≥2, history of pancreatitis	Liraglutide 0.3-3.0 mg/day SC	Placebo	BMI, adverse events	Reduction in BMI z-score (-0.28, p=0.0062), no weight changes	Liraglutide was well tolerated in children; further long-term research is needed
Kelly et al., 2020 [22]	Randomized, double-blind, placebo-controlled phase 3 clinical trial	Belgium, Mexico, Russia, Sweden, USA	2020	Evaluate the efficacy of liraglutide in reducing BMI in adolescents with obesity	12-18 years old, BMI ≥95th percentile, no response to lifestyle intervention	Type 1 diabetes, use of other obesity drugs	Liraglutide 3.0 mg/day SC for 56 weeks	Placebo	BMI, body weight, adverse events	Significant BMI reduction (-0.22 SD, p=0.002)	Liraglutide is effective in BMI reduction but with a high frequency of adverse events
Fox et al., 2025 [23]	Randomized, double-blind, placebo-controlled phase 3a clinical trial	Multicenter (23 sites in nine countries)	2025	Evaluate the efficacy of liraglutide in reducing BMI in children aged six to <12 years with obesity	Six to <12 years old, BMI ≥95th percentile, Tanner stage 1-5, no diabetes	Prior use of obesity drugs, metabolic medical conditions	Liraglutide 3.0 mg/day SC for 56 weeks	Placebo	BMI, body weight, adverse events	Significant BMI reduction (-7.4 percentage points, p<0.001)	Liraglutide reduced BMI in children but was associated with gastrointestinal adverse events

Impact on primary and secondary outcomes

Effect of Liraglutide on the BMI Z-Score

Our analysis included four RCTs [20-23] with a total of 378 subjects, out of which 211 participants were in the experimental group (liraglutide) and 167 in the control group. The values of the SMD from the individual studies ranged from -1.04 (95% CI: -1.31 to -0.78) to -0.96 (95% CI: -1.92 to 0.00), indicating a reduction in the BMI z-score in the intervention group compared to the control group.

**Figure 2:**
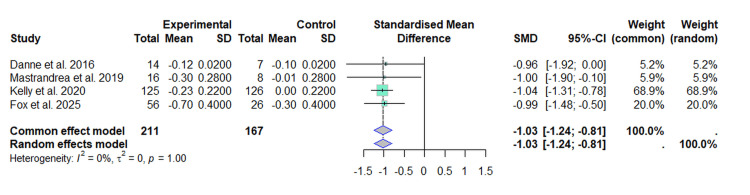
Forest plot of the effect of liraglutide on BMI (z-score) This forest plot was generated by the authors based on data extracted from the included studies [20-23].

Under the fixed-effects model, the result of the combined SMD analysis was -1.03 (95% CI: -1.24 to -0.81), while the random-effects model yielded the same result (SMD = -1.03 (95% CI: -1.24 to -0.81)) (Figure 2).

This result suggests that treatment with liraglutide is associated with a significant reduction in the BMI z-score compared to the control group. The included studies show a high consistency in their results, and there is no significant variability among them, evidenced by the null heterogeneity (I²=0%, τ²=0, p=1.00).

Effect on Metabolic Parameters: HbA1c and Fasting Glucose

**Figure 3:**
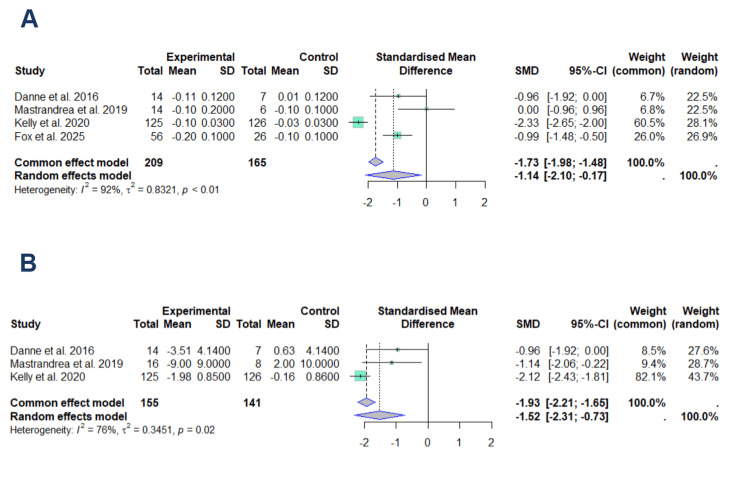
Forest plots of the effect of liraglutide on HbA1c (A) and fasting glycemia (B) This forest plot was generated by the authors based on the studies evaluating HbA1c [20-23] and fasting glycemia [20-22].

For HbA1c (Figure 3A), four studies comprising 209 subjects in the experimental group and 165 controls were included in the analysis [20-23].

The random-effects model yielded a SMD of -1.14 (95% CI: -2.10 to -0.17). High heterogeneity was observed (I²=92%, p<0.01), indicating substantial variability across studies. This heterogeneity primarily reflects the influence of studies with larger sample sizes. Notably, the study by Kelly et al. [22] contributed most significantly to the meta-analysis, representing 28.1% of the weight in the random-effects model and 60.5% in the fixed-effects model.

The second meta-analysis (Figure 3B) included three studies with 155 participants in the experimental group and 141 controls [20-22]. The random-effects model demonstrated an SMD of -1.52 (95% CI: -2.31 to -0.73), with moderate-to-high heterogeneity (I²=76%, p=0.02).

Despite the high heterogeneity in both meta-analyses, the random-effects models consistently showed a significant reduction in both HbA1c and fasting glucose levels in the experimental group compared to controls.

Total Adverse Events

**Figure 4:**
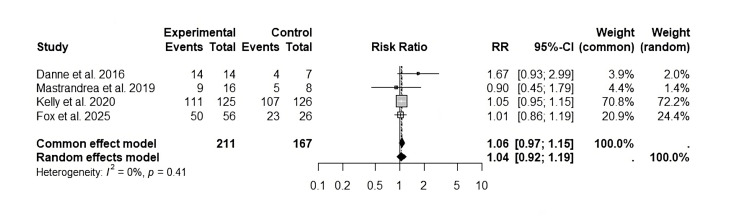
Forest plot of the adverse effects of treatment with liraglutide This forest plot was generated by the authors based on data extracted from the included studies [20-23].

Incidence of adverse effects related to the treatment included nausea, vomiting, pancreatitis, or hypoglycemia. Based on the four studies [20-23], the meta-analysis of the RR of the total adverse events is presented in Figure 4.

For the common-effects model, an RR of 1.06 (95% CI: 0.97 to 1.15) is evident, while the random-effects model estimated an RR of 1.04 (95% CI: 0.92 to 1.19) with a low variability among the included studies. There was a heterogeneity of I²=0% and p=0.41, suggesting that the estimated effects are consistent in the analyzed populations.

The results indicated that the intervention evaluated was not associated with a significant risk for total adverse events (RR=1) compared to the placebo. Furthermore, the absence of heterogeneity between the studies reinforces the robustness of the findings and suggests that the observed effects are homogeneous. Nevertheless, the predominance of a single study in the overall effect estimate should be carefully considered when interpreting the results, as it could influence the generalizability of the findings (Figure 4).

Hypoglycemia

Three studies concerning hypoglycemia were evaluated, and hypoglycemic events were reported in both cohorts. For the fixed-effects model, the RR was 1.56 [95% CI: 0.95-2.57], whereas in the random-effects model, the RR was 1.55 [95% CI: 1.00-2.40]. These findings may suggest a possible association between the intervention and an increased risk of hypoglycemia. However, the confidence interval for both the fixed- and random-effects model included unity, indicating that it was not statistically significant.

The heterogeneity analysis evidenced a value of I²=0% (p=0.85), indicating low variability between studies and suggesting that methodological differences between studies did not significantly influence the aggregate results. Within the individual analysis, Kelly et al. [22] had the highest overall effect on both models. This trend suggests that the intervention, although not statistically significant, could increase the risk of hypoglycemia, although the available data do not yet allow a definitive conclusion to be drawn.

**Figure 5:**
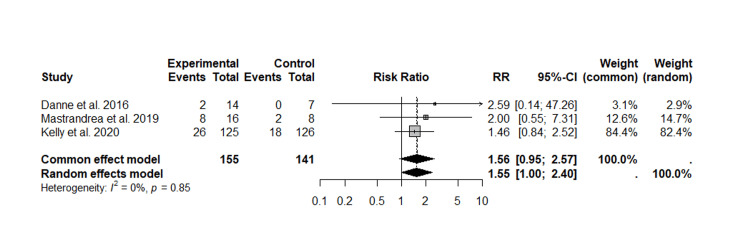
Forest plot of liraglutide treatment and hypoglycemic episodes This forest plot was generated by the authors based on data extracted from the included studies [20-22].

Given that hypoglycemia is a clinically relevant outcome due to its possible complications, these results highlight the need for further studies to provide a statistically significant conclusion in the future, with a larger sample size and longer follow-up to confirm the relationship between the intervention and the occurrence of hypoglycemic events (Figure 5).

Risk of bias in the included studies

**Figure 6:**
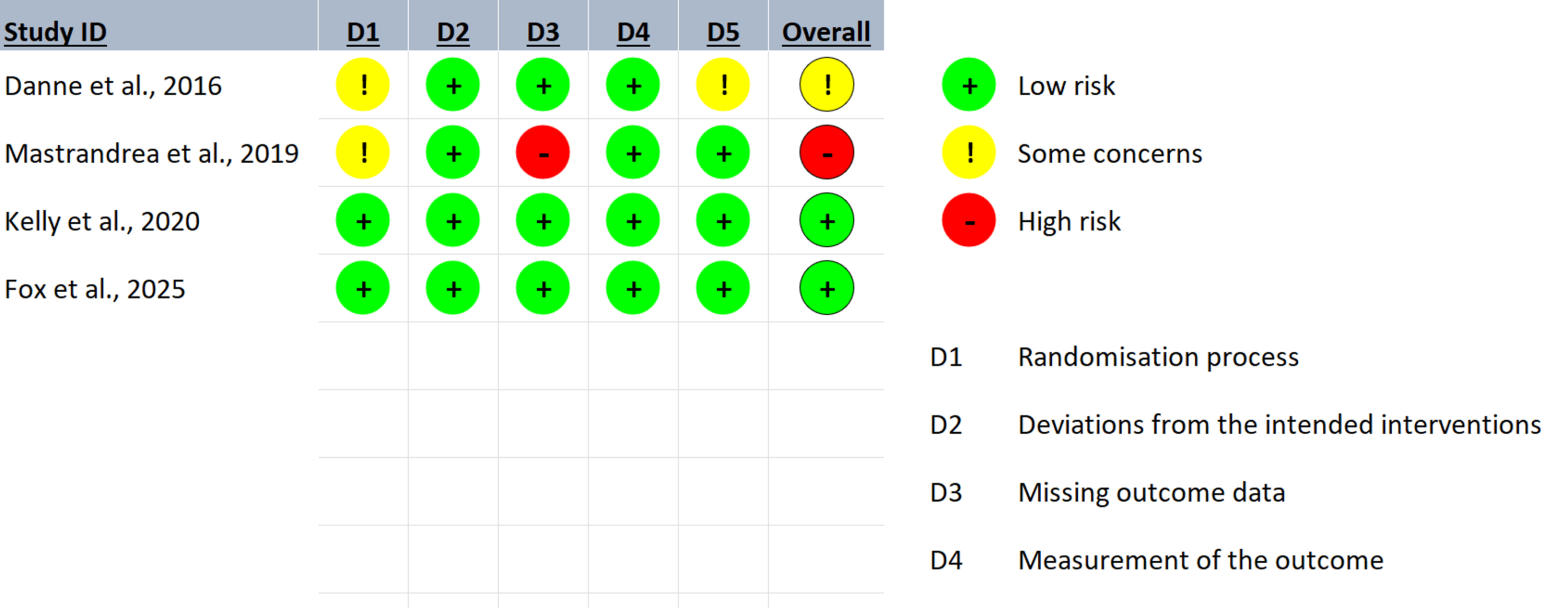
Summary of the risk of bias (RoB 2.0) [20-23]

The methodological quality of the included studies was assessed using the RoB 2.0 tool for RCTs. Figure 6 presents a graphical summary of the risk of bias in each of the five domains assessed: D1 (randomization bias), D2 (treatment deviations), D3 (missing outcome data), D4 (outcome measurement), and D5 (selection of the reported outcome).

Fifty percent of the research had a minimal risk of bias, and they received positive evaluations in every specific area. On the other hand, certain methodological limitations were found in the D1 and D3 domains [20,21]. These limitations provide evidence of some concerns regarding the risk of bias in domain D1, which may indicate deficiencies in the random sequence generation and allocation concealment, both of which are essential components to guarantee the validity of the results.

A critical aspect identified [21] was bias in D3, where one study presented a high risk of bias due to the presence of missing data, which could affect the robustness of the effect estimates. In the overall assessment, although half of the included studies present a rigorous methodology and low risk of bias, it is essential to consider the limitations identified when interpreting the results of the meta-analysis.

Sensitivity analysis

**Figure 7:**
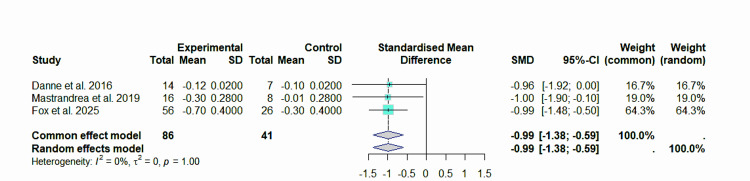
Forest plot of the effect of liraglutide on BMI (z-score), sensitivity after excluding Kelly et al. This forest plot was generated by the authors based on data extracted from the included studies [20,21,23].

To determine how reliable the conclusions are, a sensitivity analysis is carried out, which involved purposefully omitting some research from consideration. With Kelly et al. [22] removed from consideration, the pooled effect remained at -0.99 (95% CI: -1.38 to -0.59), and there was no change in heterogeneity (I²=0%), suggesting that the study did not have a major impact on the overall findings (Figure 7).

**Figure 8:**
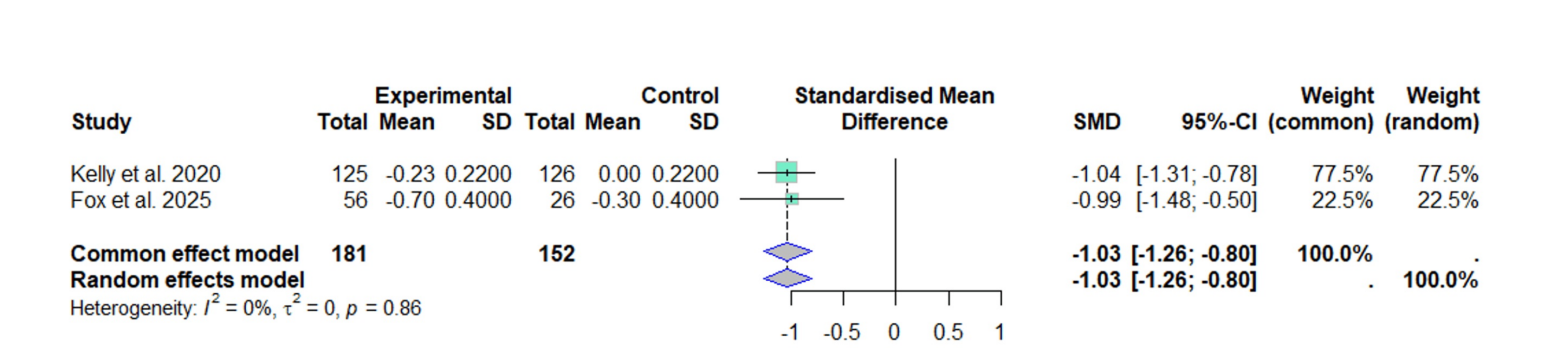
Forest plot of the effect of liraglutide on the BMI (z-score), sensitivity analysis excluding Danne et al. and Mastrandrea et al. This forest plot was generated by the authors based on data extracted from the included studies [22,23].

Similarly, the exclusion of the Danne et al. and Mastrandrea et al. [20,21] resulted in a pooled effect estimate of -1.03 (95% CI -1.26 to -0.80), with zero heterogeneity. Thus confirmed that the reduction in BMI z-score associated with liraglutide did not depend on a particular study and that the evidence was consistent (Figure 8). 

Discussion

The findings of the present investigation reveal that liraglutide is associated with a significant reduction in body weight represented by a reduction in BMI z-score, without significant evidence of an increase in hypoglycemia episodes or major side effects. However, the four clinical trials selected for the meta-analysis included 378 participants, a modest number with respect to other analyses.

Nevertheless, we highlight the relevance of this research, particularly in light of the most recent clinical trial published in 2025, whose findings contrast with previous meta-analyses on this topic [23].

We evaluated the impact of the treatment on the reduction of the BMI z-score and our findings show a SMD of -1.03, suggesting a clinically relevant reduction in patients who received liraglutide compared to the control group. The absence of heterogeneity (I^2^=0%) reinforces the robustness of these results and suggests that the magnitude of the effect is consistent across the included studies.

To evaluate these conclusions, we performed a sensitivity analysis systematically excluding Kelly et al. [22], which did not significantly alter the effect estimate. Similarly, excluding Danne et al. and Mastrandrea et al. [20,21] also resulted in a stable effect estimate. These findings suggest that the effect of liraglutide on weight reduction is consistent and does not depend on a particular study. The stability of the results reinforces the strength of the evidence and supports the validity of our conclusions.

We consider these results relevant in the context of existing literature, as previous studies have noted variability in response to liraglutide in the pediatric population with obesity [24]. However, our analysis suggests that the benefit of the drug in reducing BMI z-score is maintained even when the meta-analysis is modified. Future studies with larger numbers of participants and longer follow-up could provide more detailed insights into the sustainability of liraglutide's effect and its impact on other important metabolic outcomes.

Study limitations

Despite these positive findings, the study has some methodological limitations that should be considered when interpreting the results. First, there was a random sequencing and allocation bias (design) in some studies that could influence the comparability of the groups, introducing a potential imbalance in the baseline characteristics of the participants. This is particularly relevant in studies with small samples [20,21], where these variations in allocation can have a significant impact on the results.

On the other hand, the presence of missing data [21] introduces uncertainty about the internal validity of the results. In these cases, the lack of adherence to an intention-to-treat analysis could overestimate the effect of liraglutide on the BMI z-score reduction. Nevertheless, these studies represent approximately 5% of the weight at the time of the pooled analysis in both the fixed-effect and randomized models.

Another limitation, which should be considered when interpreting the results, is that half of the included studies were not specifically designed to evaluate weight reduction as their primary objective but focused on adverse events and pharmacodynamics of the treatment [20,21]. This represents a design bias, as the intervention may not have been administered with the same rigor or duration as the other two studies.

Finally, given the limited number of included studies (n=4), an assessment of publication bias using a funnel plot or under Egger's statistic would not be appropriate. However, we consider that does not invalidate the feasibility of the meta-analysis, as the consistency of our results and the low heterogeneity in the primary objective support the pooled estimator. Nevertheless, the inclusion of new studies in the future with a more robust design and larger sample size would improve the precision of the estimates and strengthen the external validity of our findings.

## Conclusions

This meta-analysis suggests that liraglutide could be a useful therapeutic option in pediatric obesity, especially in patients who have not achieved significant weight reduction with conventional interventions. The BMI z-score reduction observed in this analysis, together with the absence of heterogeneity between studies, supports its efficacy. However, its implementation should be individualized, considering the potential adverse effects, and rigorous monitoring should be carried out to ensure safety. Considering the limited number of studies that are currently available, more research with a bigger sample size and a longer period of follow-up is necessary to examine the long-term effects of liraglutide in pediatric populations, as well as its influence on other metabolic outcomes such as insulin resistance. Evaluation of a combination with behavioral and lifestyle treatments should also be included in future studies to maximize the effectiveness of long-term results.
